# Revisional Surgery of One Anastomosis Gastric Bypass for Severe Protein–Energy Malnutrition

**DOI:** 10.3390/nu14112356

**Published:** 2022-06-06

**Authors:** Adam Abu-Abeid, Or Goren, Shai Meron Eldar, Antonio Vitiello, Giovanna Berardi, Guy Lahat, Danit Dayan

**Affiliations:** 1 Division of General Surgery, Tel Aviv Sourasky Medical Center, 6 Weizman Street, Tel Aviv 6423906, Israel; shaime@tlvmc.gov.il (S.M.E.); guyla@tlvmc.gov.il (G.L.); danitd.75@gmail.com (D.D.); 2 Sackler’s Faculty of Medicine, Tel-Aviv University, Tel Aviv 6997801, Israel; goren.orr@gmail.com; 3 Division of Anesthesiology, Pain and Intensive Care, Tel Aviv Medical Center, Tel Aviv 6423906, Israel; 4 Advanced Biomedical Sciences Department, Naples “Federico II” University, AOU “Federico II”—Via S. Pansini 5, 80131 Napoli, Italy; antoniovitiello_@hotmail.it (A.V.); giovannaberardi88@gmail.com (G.B.)

**Keywords:** one anastomosis gastric bypass, protein, malnutrition, revisional surgery, reversal, conversion, bariatric metabolic surgery

## Abstract

Background: One anastomosis gastric bypass (OAGB) is safe and effective. Its strong malabsorptive component might cause severe protein–energy malnutrition (PEM), necessitating revisional surgery. We aimed to evaluate the safety and outcomes of OAGB revision for severe PEM. Methods: This was a single-center retrospective analysis of OAGB patients undergoing revision for severe PEM (2015–2021). Perioperative data and outcomes were retrieved. Results: Ten patients underwent revision for severe PEM. Our center’s incidence is 0.63% (9/1425 OAGB). All patients were symptomatic. Median (interquartile range) EWL and lowest albumin were 103.7% (range 57.6, 114) and 24 g/dL (range 19, 27), respectively, and 8/10 patients had significant micronutrient deficiencies. Before revision, nutritional optimization was undertaken. Median OAGB to revision interval was 18.4 months (range 15.7, 27.8). Median BPL length was 200 cm (range 177, 227). Reversal (*n* = 5), BPL shortening (*n* = 3), and conversion to Roux-en-Y gastric bypass (RYGB) (*n* = 2) were performed. One patient had anastomotic leak after BPL shortening. No death occurred. Median BMI and albumin increased from 22.4 kg/m^2^ (range 20.6, 30.3) and 35.5 g/dL (range 29.2, 41), respectively, at revision to 27.5 (range 22.2, 32.4) kg/m^2^ and 39.5 g/dL (range 37.2, 41.7), respectively, at follow-up (median 25.4 months, range 3.1, 45). Complete resolution occurs after conversion to RYGB or reversal to normal anatomy, but not after BPL shortening. Conclusions: Revisional surgery of OAGB for severe PEM is feasible and safe after nutritional optimization. Our results suggest that the type of revision may be an important factor for PEM resolution. Comparative studies are needed to define the role of each revisional option.

## 1. Introduction

One anastomosis gastric bypass (OAGB) is the third most commonly performed bariatric metabolic surgery (BMS) worldwide [1], and the most common in our bariatric center. It combines restriction with a dominant malabsorptive component, and it is simple to perform, with a short learning curve [2,3]. OAGB is considered safe and effective as both primary and secondary BMS. It confers good outcomes in terms of satisfactory weight reduction and resolution of obesity-associated medical problems [4,5,6]. OAGB results in equal or better outcomes compared with Roux-en-Y gastric bypass (RYGB) on the one hand and more nutritional deficiencies on the other hand, owing to its stronger malabsorptive component [7,8], i.e., the longer biliopancreatic limb (BPL). 

Severe protein–energy malnutrition (PEM) is an uncommon complication following OAGB that rarely requires corrective surgery [9] like in other BMS [10]. There is no uniform definition in the bariatric literature for protein–energy malnutrition, its severity grading or threshold for revisional surgery. 

We aimed to evaluate the safety and outcomes of revisional surgery for severe PEM.

## 2. Materials and Methods

A retrospective review of a single bariatric center database, and the hospital computerized medical record system were performed. Ten OAGB patients reoperated at our medical center (January 2015 to December 2021) due to severe PEM were included in this study. Nine of them underwent the OAGB in our medical center. In the period corresponding to the study, 1425 cases of OAGB were performed in our bariatric center. Prior to OAGB, all patients were found eligible for BMS by the multidisciplinary bariatric team, according to the American Society for Metabolic and Bariatric Surgery (ASMBS) guidelines. 

Before revisional surgery, all patients underwent a thorough workup by a multidisciplinary team, including psychological and/or psychiatric evaluation, nutritional assessment, anthropometric studies (weight, height, body mass index (BMI)) laboratory tests, gastrointestinal investigation (upper and lower gastrointestinal endoscopy, computed tomography, barium swallow test, abdominal ultrasound). All patients received nutritional support, including enteral and/or total parenteral nutrition and micronutrient supplements. High-dose Loperamide and Pancreolipase were given to 8/10 and 7/10 patients, respectively. All patients gave their written informed consent, following elaborate explanations about the indication for reoperation, surgical options, possible complications, surgical re-interventions, and implications on future bariatric and metabolic outcomes.

Our threshold for revisional surgery: we defined severe PEM as the criterion for revision. 

Our definition of severe PEM: significant symptoms of hypoalbuminemia, such as edema, and weakness, with or without micronutrient deficiency, that persisted or recurred despite nutritional optimization. 

Significant micronutrient deficiency: moderate-severe deficiency of iron, calcium, vitamin D3, B1, B6, B12, and folic acid [11]. 

Data retrieved at both surgeries (OAGB and reoperation) included demographic information, clinical characteristics, work-up findings, nutritional parameters and intervention, medical treatment, operative information, and postoperative outcomes. Early (30-day) and late (>30-day) complications were graded according to the Clavien–Dindo (CD) classification [12].

### 2.1. Statistical Analysis

Continuous data are presented as medians (interquartile range (IQR)). Proportions are presented as *n* (%). Dichotomous data were analyzed using the Fisher’s exact test. Continuous data were analyzed using the Mann–Whitney test.

### 2.2. Revisional Surgery Technique Highlights

1. Reversal to normal anatomy:
  a. Horizontal antimesenteric transection of the gastro-jejunostomy, while preserving adequate intestinal continuity, using a linear stapler. The old staple line is then resected off the distal pouch.
  b. Construction of wide side-to-side gastro-gastrostomy, using a 60 mm cartridge linear stapler and a continuous self- retaining suture for defect closure.
2. BPL shortening:
  a. Horizontal antimesenteric transection of the gastro-jejunostomy, while preserving adequate intestinal continuity, using a linear stapler. The old staple line is then resected off the distal pouch.
  b. Refashioning of the BPL length at 65, 100, and 120 cm (from 170, 170, and 220 cm, respectively) and the creation of a new side-to-side gastro-jejunostomy.
3. Conversion to RYGB:
  a. Resection of the gastro-jejunal complex, using 3 firings of a linear stapler.
  b. Re-establishment of bowel continuity by construction of side-to-side jejuno-jejunostomy.
  c. Conversion to RYGB, with a BPL length of 50 and 100 cm (instead of 350 and 200 cm, respectively).

The study was approved by the Tel-Aviv Sourasky Medical Center Institutional Review Board (TLV-16-0325/2019) and was performed in accordance with the ethical standards of the institutional and/or national research committee and with the 1964 Declaration of Helsinki and its later amendments or comparable ethical standards. Informed consent was obtained from all individual participants included in this study. 

## 3. Results

Ten patients underwent revisional surgery due to severe PEM. The incidence of revisional surgery for PEM in our bariatric center is only 0.63% (9/1425 OAGB). Patients` characteristics at OAGB are presented in Table 1. The male-to-female ratio was 2:8, the median (interquartile range) age was 47.5 years (range 42.2, 58.2), and the median BMI was 44.2 kg/m^2^ (range 35.8, 47). The OAGB was primary or secondary in five patients each. Previous BMS included sleeve gastrectomy, adjustable gastric banding, and silastic ring vertical gastroplasty (*n* = 2, 2, and 1, respectively). The median BPL length was 200 cm (range 177, 200). All patients were in good nutritional status before undergoing OAGB, and 3/10 had mild micronutrient deficiency (iron, B12, or vitamin D).

The patients’ characteristics of PEM are presented in Table 2. The median EWL was 103.7% (range 57.6, 114), and the median TWL was 43% (range 35.7, 56.9). Symptoms included mainly marked weakness, dizziness, syncope, peripheral edema, and diarrhea (watery or steatorrhea). The lowest albumin was median 24 g/dL (range 19, 27), and 8/10 patients had >1 significant vitamin/ mineral deficiencies (iron, calcium, vitamin D3, B1, B6, B12, folic acid). Nutritional optimization included nutritional supplements and medications to aid absorption, consisting of enteral nutrition (EN), total parenteral nutrition (TPN), multivitamins (MV), loperamide, and pancreolipase (*n* = 10, 5, 10, 4, 8, and 7, respectively).

The patients’ characteristics at revisional surgery are presented in Table 3. Revision was performed at a median interval of 18.4 months (range 15.7, 27.8) after OAGB. At revision, the median age, BMI, and highest albumin were 49.5 years (range 43.5, 61.5), 22.4 kg/m^2^ (range 20.6, 30.3), and 35.5 g/dL (range 29.2, 41), respectively. The type of revision included reversal to normal anatomy, BPL shortening, and conversion to RYGB, performed in five, three, and two patients, respectively. Revisional surgery was laparoscopic in 8/10 patients, and through laparotomy in 2/10 patients (one reversal and another conversion to RYGB). BPL was found longer than reported in 2/10 patients (250 instead of 200, and 350 instead of 250 cm), and the median actual BPL length was 200 cm (range 177, 227). At revision, the common channel was found of at least 300 cm, except one, who was operated on at another hospital for the OAGB. The median operative time was 88.5 min (range 66.8, 117). The median LOS was 8 days (range 7.2, 12.2) and early complications CD >3 occurred in one patient (1/10), who had an anastomotic leak after BPL shortening and underwent debridement with primary repair. There was no occurrence of mortality. None of the patients had liver function derangements. None of the patients had villous atrophy or other gastrointestinal tract pathologies on pre-revision investigation (0/10) or in examined operative specimen (0/6). 

The median follow-up after revision was 25.4 months (range 3.1, 45). The median weight, BMI, and albumin increased from 60 kg (range 51.7, 79.2), 22.4 kg/m^2^ (range 20.6, 30.3), and 35.5 g/dL (range 29.2, 41), respectively, at revision to 73.5 kg (range 59, 89), 27.5 kg/m^2^ (range 22.2, 32.4), and 39.5 g/dL (range 37.2, 41.7), respectively, at last follow-up (median 25.4 months, range 3.1, 45). PEM has resolved in 7/10 patients, improved in 2/10 still on supportive medications for PEM and nutritional support, and did not change in 1/10. The latter had undergone BPL shortening and is a candidate for reversal to normal anatomy. BPL shortening did not result in complete resolution, as opposed to conversion to RYGB and reversal. In a univariate analysis including all of the aforementioned parameters, we found that only the type of revision had a significant correlation with the complete resolution of PEM. Prior to revision, all patients had a BPL length >150 cm, and in a univariate analysis, none of the variables were found as a risk factor for the development of PEM. The BMS effects were maintained in 9/10 patients, whereas 1/10 patients regained weight significantly to BMI >40 kg/m2. All the patients that had T2D (*n* = 3) and NAFLD (*n* = 4) at OAGB had a complete resolution at revisional surgery and last follow-up.

## 4. Discussion

OAGB is one of three commonly performed, acceptable BMSs [13]. There is cumulative evidence of satisfactory excess weight loss—88% at 2 years, 77% at 6 years, and 70% at 12 years postoperatively [6]—and remission or improvement of obesity associated medical problems, especially type II diabetes (T2D) resolution—85–90% at 1 year postoperatively [4,14] and ~70% at 5–15 years [15]. OAGB is safe, with 5% overall morbidity and 0.2% mortality rates [5]. However, as in other BMSs, worrisome, delayed onset complications might develop and affect patients’ health and quality of life. These include mainly bile reflux, anastomotic ulcer, and PEM and may infrequently require revisional surgery [16]. Khrucharoen et al. showed in a systematic review that 26% (46/179) of OAGB revisions were for PEM [17]. 

The incidence of revisions for PEM is variable between different studies. Rutledge reported that 31/2410 patients (1.28%) underwent reversal due to excessive weight loss [18]. Parmar et al. found a cumulative rate of 0.71% (range, 0–3.8%) in a systematic review of 12,807 OAGB patients [19]. Recent studies, such as the Italian multi-institutional survey of Musella et al. [16] and the single center studies of Jedamzik et al. [20] and Almuhanna et al. [15], reported rates of 0.18% (16/8676 OAGB patients), 0.9% (9/1025 OAGB patients), and 2.3% (51/2223 OAGB patients), respectively. In the current single-center study, the rate is only 0.63% (9/1425 OAGB). In-line with the literature, most of our patients (7/10) had their OAGB to revision interval within the second postoperative year. However, it can develop thereafter. We encourage more surgeons to report their long-term rates of this uncommon complication. 

PEM after OAGB is mainly attributed to its strong malabsorptive component, i.e., the BPL length. In a comparative study of BPL lengths of 150, 180, and 250 cm, Ahuja et al. [21] found no significant difference in OAGB effectiveness at 1 year (i.e., EWL, resolution of T2D, HTN); however, 250 cm BPL was associated with worse nutritional deficiencies, and one patient died of liver failure. In another comparative study of BPL lengths of 150, 180, and 200 cm, Pizza et al. [22] did not find any significant difference in effectiveness or nutritional status at 2 years, except for iron and ferritin levels, which were significantly lower in 200 cm BPL. Nevertheless, in both studies, none of the patients underwent revisional surgery. In contrast, the current study focuses on patients operated for severe PEM, and none of them had a BPL length of 150 cm (median 200 cm, range 177, 227). In 2/10 patients, the actual BPL found at revision was longer than reported at OAGB. We therefore advise to count the BPL length out loud during OAGB, for team double-checking. We do not routinely measure the total small bowel length (SBL) at the OAGB. In the first years, we used a fixed ~200 cm BPL and occasionally a 250 cm-BPL for BMI > 60 without counting the total SBL. Following accumulating data and personal experience, though uncommon occurrence of significant diarrhea, excessive weight loss, and nutritional deficiencies, we changed the BPL length into 150–200 cm, depending mainly on the patient’s BMI, and indication (primary or secondary OAGB). Ramos et al. have outlined the 2020 IFSO consensus conference statement on OAGB, postulating a clear consensus that BPL length <200 cm was adequate, BPL should only be >200 cm if total SBL is measured and suitably long, and that BPL length can be determined according to the BMI [23]. Total SBL varies widely [24]; therefore, some surgeons routinely measure the total SBL and tailor the BPL accordingly, in order to gain adequate bariatric and metabolic goals on the one hand and minimize PEM occurrence on the other hand. Komaei et al. [25] retrospectively compared outcomes between fixed 200 cm BPL without SBL measurements to tailored BPL of 40% from total SBL. There was no significant difference in terms of efficacy, but more patients in the fixed BPL group had nutritional deficiencies (*p* < 0.05), none of them necessitated revision. Many surgeons have not endorsed this approach, and there is no consensus regarding its necessity or the ideal BPL percentage of total SBL [23]. Furthermore, we share the fear of other bariatric surgeons of adding a risk of unintentional bowel injury as an argument against routine total SBL measurements. Instead, we believe such measurements are important at revisional surgery of OAGB in cases of severe PEM or following weight regain (WR) when considering malabsorption intensification as in the case of RYGB [26]. 

Projecting from WR, the etiology of PEM is probably multifactorial [26]. An altered gut–hormonal balance may explain cases of PEM that are refractory to intensive treatment and cases of ongoing PEM despite BPL shortening. Further investigation is needed to support or refute our assumption. New-onset anorexia or pre-existing psychiatric disorders are other possible causes. Some patients do not comply with the strict nutritional recommendations, perhaps due to their high cost, even in the event of PEM. Secondary pathologies such as colitis, enteritis with atrophy of the villi, undiagnosed celiac disease, pancreatic insufficiency, or small intestinal bacterial overgrowth could serve as another explanation. In our cohort, all patients were in good nutritional status before undergoing OAGB, and none of them had any evidence of the aforementioned GI pathologies, but they were non-compliant with nutritional recommendations after OAGB. Revisional surgery for PEM should be preceded by nutritional status optimization, which is also partly dependent on patients’ compliance. 

There are several revision options described in the literature, including BPL shortening, conversion to RYGB, conversion to SG, and reversal to normal anatomy. All these surgical options are generally feasible and relatively safe. However, PEM-wise, it is difficult to assess the cumulative complication and resolution rates for each revision option. This is for several reasons. Most studies do not focus on PEM as a sole indication for revision. The complication rate of revision for PEM may be higher than for other indications, especially if not amenable for optimal correction prior to revision, as occurred in one of our patients. In addition, most reports do not elaborate on PEM outcomes after revision, and some studies did not specify which revision types were performed. Furthermore, there is lack of standardized criteria for revisional surgery, and most studies do not specify their definition of severe PEM, and rather simply use the terms: ‘malnutrition’, ‘protein–energy/calorie malnutrition’, ‘excessive weight loss’, ‘macro or micro—nutrient deficiency’, ‘hypoalbuminemia’, etc. There is no standardized protocol of nutritional status optimization prior to revision either. In addition, there is variance regarding BPL lengths and other OAGB parameters. Henceforth, it is difficult to assess the best revisional surgery for PEM or delineate guidelines or algorithm for the management of severe PEM after OAGB. 

Despite these limitations, few observations can be made regarding the different revisional options. Reversal of OAGB is generally feasible, safe, and simple to perform [27]. Gesner et al. [28] reported a high rate of overall morbidity, which was reduced when they switched from simple transection of the gastro-jejunostomy to resection and construction of jejuno-jejunostomy (50 vs. 8.3%, *p* = 0.03). In their first 14 cases, they left the old gastro-jejunal staple line untouched and consequently had 4 complications there (leak and stenosis), while in the current study, although we had fewer patients, we prevented these complications (0/5 patients) by resecting the old staple line and assuring adequate patency of the intestinal lumen. We therefore suggest to beware of two pitfalls: First, simple transection of the gastro-jejunal anastomosis should be performed only if it is possible to preserve an adequate intestinal patency (i.e., without narrowing the lumen) and provided the old staple line is completely resected. Otherwise, resection with jejuno-jejunal anastomosis is advised. Second, gastro-gastrostomy should be wide enough (>6 cm) to prevent stricture. Reversal to normal anatomy is advantageous since it ensures resolution of PEM, and we believe it is more adequate for patients with severe PEM, especially non-compliant ones. The disadvantage of reversal is the possible risk of significant weight regain and recurrence of obesity-associated problems. Consequently, many patients refuse this option and prefer one of the revisional alternatives, which are more likely to preserve BMS outcomes. 

BPL shortening maintains the ‘OAGB structure’ while reducing the malabsorptive component and therefore is supposed to result in PEM resolution. Interestingly, while Hussain et al. [29] reported the resolution of intractable diarrhea, PEM, or deranged liver functions in eight patients undergoing BPL shortening to 150 cm (from >200 cm), none of our three patients had resolution of PEM, despite a shorter revised BPL of 65–120 cm (from 170 or 220 cm). We assume that for longer initial BPL, shortening may sometimes be adequate; however, this revisional option mandates careful surveillance due to the risk of ongoing PEM. More data are needed. 

Conversion to RYGB or SG are other options that are likely to preserve BMS outcomes. Chen et al. [30] performed conversion to sleeve gastrectomy by transecting the gastro-jejunal anastomosis, applying hand-sewn anastomosis of the distal gastric pouch to the antrum of the remnant stomach and vertical resection of the remnant stomach over an endoscope. They reported significant improvement in malnutrition with a maintained weight loss at early follow-up. Their study included, however, both OAGB and RYGB patients and other indications for revision, and the overall complication rate was 8.1% for the entire cohort. We did not perform this type of revisional surgery because of the possibility to replace PEM with other complications of SG (for example, gastroesophageal reflux disease or stricture) and because it prevents future reversal to normal anatomy in case of persistent nutritional deficiencies. Jedamzik et al. performed conversions to RYGB and concluded that BPL shortening to 35–100 cm with conversion to RYGB is feasible and safe for severe malnutrition [20]. Similarly, two of our patients were converted to RYGB with 50–100 cm revised BPL and achieved PEM resolution. 

Khrucharoen et al. [17] concluded that although revision to RYGB was technically simpler than revision to SG or normal anatomy, it should be avoided in PEM, due to the risk of further malabsorption posed by the remaining bypassed BPL. They also mentioned that a three-limb measurement is important to decide if we need to relocate the gastrojejunal anastomosis or simply create the jejunojejunostomy, since the latter option might exacerbate malnutrition. They also concluded that reversal to original anatomy is beneficial, though can be technically challenging and may be associated with an increased risk of complications (i.e., gastrojejunal leak and stenosis). Haddad et al. [1] reported in the IFSO worldwide OAGB survey the operative data of 239/277 patients that underwent revision for malnutrition or steatorrhea. Revisions included: conversion to RYGB (43%), reversal (32%), BPL shortening (20%), and conversion to SG (5%). BPL length data were available in 244/277 patients, revealing that, most commonly, the BPL length in these patients was 200 cm. They also reported a 5% (5/98 OAGB mortalities) mortality rate due to liver failure or malnutrition. Kermansaravi [31] reported a cumulative rate of revisions for PEM of 0.84% (153/17,938 patients) and suggested avoiding creating a BPL >150 cm to reduce the rates of PEM, given that BMS outcomes seem similar for 150 cm compared with 200 cm [32]. In the current study, we found the revision type is an important factor for complete resolution of PEM; however, large comparative studies are needed to examine all aspects of revisional surgery for severe PEM following OAGB. 

Our study has several limitations. This is a retrospective observational study of a small cohort and no comparative groups. The real incidence of PEM may be underestimated, due to a ~30% ‘loss to follow-up’. Large comparative studies are needed to appreciate the proper surgical management of severe PEM, to delineate uniform threshold for revisional surgery, and to identify risk factors for the development of PEM.

Despite these limitations, this is a very important issue for further discussion and investigation since it might rarely lead to hepatic insufficiency and death. We have described in detail this uncommon complication and outcomes of three different revisional options. We believe that OAGB is a very good surgery to treat severe obesity, and it is associated with uncommon occurrence of delayed complications occurring in other BMS. 

## 5. Conclusions

Revisional surgery of OAGB for severe PEM is feasible and safe after nutritional optimization. Our results suggest that the type of revision may be an important factor for PEM resolution. Comparative studies are needed to better define the role of each revisional option. 

## Figures and Tables

**Table 1:** Perioperative characteristics of OAGB.

Patient	Gender	Age (Years)	BMI (kg/m^2^)	Previous Bariatric Procedure	Associated Medical Problems	Recorded BPL Length (cm)	30-Day Complications	LOS (Days)
1	F	71	45.1	-	T2D, HTN, HL, NAFLD	200	-	2
2	M	46	39.1	-	Smoking	200	-	2
3	F	46	46.4	AGB	NAFLD, OA	170	-	2
4	F	50	45.1	AGB	-	220	-	4
5	F	58	35.3	-	T2D, HL	200	-	2
6	M	44	48.9	SG	T2D, HTN, HL, Smoking	200	-	5
7	F	37	43.3	-	-	170	-	2
8	F	59	60.9	SG	NAFLD, OA	250	-	3
9 *	F	49	35.0	SRVG	Smoking	200	Anastomotic leak	80
10	F	22	35.6	-	NAFLD	180	-	3

OAGB—One anastomosis gastric bypass; BMI—Body mass index; BPL—Biliopancreatic limb; LOS—Length of stay; AGB—Assisted gastric band; SG—Sleeve gastrectomy; SRVG—Silastic ring vertical gastroplasty; T2D—Type 2 diabetes; HTN—Hypertension; HL—Hyperlipidemia; NAFLD—Non-alcoholic fatty liver disease; OA—Osteoarthritis. * The OAGB was performed in another hospital.

**Table 2:** Preoperative characteristic of OAGB patients with severe PEM.

Patient	%EWL	%TWL	Symptoms General Stool Frequency (/Day) and Consistency		Lowest Albumin Level (g/dL)	Significant Vitamin/Mineral Deficiencies	Nutritional Optimization
1	53.1	29	Marked weakness, perioral paresthesia,	3, normal	24	D_3_, Ca^2+^	EN, MV
2	122.8	56.5	Marked weakness, dizziness	3, steatorrhea	26	B_1_, Fe^2+^, D_3,_ Ca^2+^	EN, MV, Loperamide, Pancrease
3	58.4	32.8	Marked weakness	20, watery	34	-	EN, MV, Loperamide, Pancrease
4	72.3	39.2	Syncope, falls, peripheral edema	8, watery	21	B_1_, D_3_, Fe^2+^	TPN, EN, MV, Loperamide, Pancrease
5	102.5	42.1	Marked weakness, syncope, dizziness, peripheral edema	10, steatorrhea	15	Fe^2+^, Ca^2+^	TPN, EN, MV, Loperamide, Pancrease
6	109.7	58.1	-	5, steatorrhea	28	D_3_	EN, MV, Loperamide, Pancrease
7	111.1	60	Marked weakness, limb pain, peripheral edema	6, steatorrhea	16	B_1_, B_6_, B_12_, Folate, D_3_, Ca^2+^, Fe^2+^	TPN, EN, MV, Loperamide, Pancrease
8	55	36.7	Syncope, weakness, peripheral edema	6, watery	20	B_1_, D_3_, Ca^2+^, Fe^2+^	TPN, EN, MV, Loperamide
9	126.3	51.6	Marked weakness, dizziness, palpitations, peripheral edema	6, watery	24	B_1_, Fe^2+^	TPN, EN, MV, Loperamide, Pancrease
10	104.9	43.9	Marked weakness	2, normal	27	-	EN, MV

OAGB—One anastomosis gastric bypass; PEM—Protein–energy malnutrition; EWL—Excess weight loss; TWL—Total weight loss; MV—Multivitamin; EN—Enteral nutrition; TPN—Total parenteral nutrition.

**Table 3:** Perioperative data of OAGB revision.

Patient	Age (Years)	Time from OAGB to Revision (Months)	BMI at Day of Revision (kg/m^2^)	Albumin at Day of Revision (g/dL)	Actual BPL Length (cm)	Type of OAGB Revision	Operative Time (Minutes)	30-Day Complications	LOS (Days)
1	72	15	22.5	36	200	Reversal—Gastro-gastrostomy	60	-	4
2	48	25	22.4	35	200	Reversal—Gastro-gastrostomy	59	-	5
3	47	16	31.2	41	170	BPL shortening to 100 cm	77	-	8
4	53	38	27.5	41	220	BPL shortening to 120 cm	129	-	11
5	63	61	19.3	37	250	Reversal—Gastro-gastrostomy	70	-	16
6	45	17	30	41	200	Reversal—Gastro-gastrostomy	100	-	8
7	39	18	18.7	22	170	BPL shortening to 65 cm	69	Anastomotic leak	81
8	61	22	41.5	30	350	Conversion to RYGB—BPL 50 cm	155	Urinary tract infection	10
9	51	24	21	30	200 *	Conversion to RYGB—BPL 100 cm	70	-	8
10	24	19	21	27	180	Reversal—Gastro-gastrostomy	113	-	8

OAGB—One anastomosis gastric bypass; BMI—Body mass index; BPL—Biliopancreatic limb; LOS—length of stay; BPL—Biliopancreatic limb; RYGB—Roux-en-Y gastric bypass. * Efferent limb of 100 cm.

## References

[1] Haddad A., Bashir A., Fobi M., Higa K., Herrera M.F., Torres A.J., Himpens J., Shikora S., Ramos A.C., Kow L. (2021). The IFSO Worldwide One Anastomosis Gastric Bypass Survey: Techniques and Outcomes?. Obes. Surg..

[2] Felsenreich D.M., Bichler C., Langer F.B., Gachabayov M., Eichelter J., Gensthaler L., Vock N., Artemiou E., Prager G. (2020). Surgical Technique for One-Anastomosis Gastric Bypass. Surg. Technol. Int..

[3] Lo H.C. (2020). The learning curve of one anastomosis gastric bypass and its impact as a preceding procedure to Roux-en Y gastric bypass: Initial experience of one hundred and five consecutive cases. BMC Surg..

[4] Abu-Abeid A., Lessing Y., Pencovich N., Dayan D., Klausner J.M., Abu-Abeid S. (2018). Diabetes resolution after one anastomosis gastric bypass. Surg. Obes. Relat. Dis..

[5] Chevallier J.M., Arman G.A., Guenzi M., Rau C., Bruzzi M., Beaupel N., Zinzindohoué F., Berger A. (2015). One thousand single anastomosis (omega loop) gastric bypasses to treat morbid obesity in a 7-year period: Outcomes show few complications and good efficacy. Obes. Surg..

[6] Carbajo M.A., Luque-de-León E., Jiménez J.M., Ortiz-de-Solórzano J., Pérez-Miranda M., Castro-Alija M.J. (2017). Laparoscopic One-Anastomosis Gastric Bypass: Technique, Results, and Long-Term Follow-Up in 1200 Patients. Obes. Surg..

[7] Bhandari M., Nautiyal H.K., Kosta S., Mathur W., Fobi M. (2019). Comparison of one-anastomosis gastric bypass and Roux-en-Y gastric bypass for treatment of obesity: A 5-year study. Surg. Obes. Relat. Dis..

[8] Magouliotis D.E., Tasiopoulou V.S., Tzovaras G. (2018). One anastomosis gastric bypass versus Roux-en-Y gastric bypass for morbid obesity: A meta-analysis. Clin. Obes..

[9] De Luca M., Piatto G., Merola G., Himpens J., Chevallier J.M., Carbajo M.A., Mahawar K., Sartori A., Clemente N., Herrera M. (2021). IFSO Update Position Statement on One Anastomosis Gastric Bypass (OAGB). Obes. Surg..

[10] Lange J., Königsrainer A. (2019). Malnutrition as a Complication of Bariatric Surgery—A Clear and Present Danger?. Visc. Med..

[11] Mechanick J.I., Apovian C., Brethauer S., Garvey W.T., Joffe A.M., Kim J., Kushner R.F., Lindquist R., Pessah-Pollack R., Seger J. (2020). Clinical practice guidelines for the perioperative nutrition, metabolic, and nonsurgical support of patients undergoing bariatric procedures—2019 update: Cosponsored by American Association of Clinical Endocrinologists/American College of Endocrinology, The Obesity Society, American Society for Metabolic & Bariatric Surgery, Obesity Medicine Association, and American Society of Anesthesiologists. Surg. Obes. Relat. Dis..

[12] Clavien P.A., Barkun J., de Oliveira M.L., Vauthey J.N., Dindo D., Schulick R.D., de Santibañes E., Pekolj J., Slankamenac K., Bassi C. (2009). The Clavien-Dindo classification of surgical complications: Five-year experience. Ann. Surg..

[13] Di Lorenzo N., Antoniou S.A., Batterham R.L., Busetto L., Godoroja D., Iossa A., Carrano F.M., Agresta F., Alarçon I., Azran C. (2020). Clinical practice guidelines of the European Association for Endoscopic Surgery (EAES) on bariatric surgery: Update 2020 endorsed by IFSO-EC, EASO and ESPCOP. Surg. Endosc..

[14] Musella M., Apers J., Rheinwalt K., Ribeiro R., Manno E., Greco F., Čierny M., Milone M., Di Stefano C., Guler S. (2016). Efficacy of Bariatric Surgery in Type 2 Diabetes Mellitus Remission: The Role of Mini Gastric Bypass/One Anastomosis Gastric Bypass and Sleeve Gastrectomy at 1 Year of Follow-up. A European survey. Obes. Surg..

[15] Almuhanna M., Soong T.C., Lee W.J., Chen J.C., Wu C.C., Lee Y.C. (2021). Twenty years’ experience of laparoscopic 1-anastomosis gastric bypass: Surgical risk and long-term results. Surg. Obes. Relat. Dis..

[16] Musella M., Vitiello A., Susa A., Greco F., De Luca M., Manno E., Olmi S., Raffaelli M., Lucchese M., Carandina S. (2022). Revisional Surgery After One Anastomosis/Minigastric Bypass: An Italian Multi-institutional Survey. Obes. Surg..

[17] Khrucharoen U., Juo Y.Y., Chen Y., Dutson E.P. (2020). Indications, operative techniques, and outcomes for revisional operation following mini-gastric bypass-one anastomosis gastric bypass: A systematic review. Obes. Surg..

[18] Rutledge R., Walsh T.R. (2005). Continued excellent results with the mini gastric bypass: Six-year study in 2,410 patients. Obes. Surg..

[19] Parmar C.D., Mahawar K.K. (2018). One anastomosis (mini) gastric bypass is now an established bariatric procedure: A systematic review of 12,807 patients. Obes. Surg..

[20] Jedamzik J., Bichler C., Felsenreich D.M., Gensthaler L., Eichelter J., Nixdorf L., Krebs M., Langer F.B., Prager G. (2022). Conversion from one-anastomosis gastric bypass to Roux-en-Y gastric bypass: When and why-a single-center experience of all consecutive OAGB procedures. Surg. Obes. Relat. Dis..

[21] Ahuja A., Tantia O., Goyal G., Chaudhuri T., Khanna S., Poddar A., Gupta S., Majumdar K. (2018). MGB-OAGB: Effect of Biliopancreatic Limb Length on Nutritional Deficiency, Weight Loss, and Comorbidity Resolution. Obes. Surg..

[22] Pizza F., Lucido F.S., D’Antonio D., Tolone S., Gambardella C., Dell’Isola C., Docimo L., Marvaso A. (2020). Biliopancreatic Limb Length in One Anastomosis Gastric Bypass: Which Is the Best?. Obes. Surg..

[23] Ramos A.C., Chevallier J.M., Mahawar K., Brown W., Kow L., White K.P., Shikora S., IFSO Consensus Conference Contributors (2020). IFSO (International Federation for Surgery of Obesity and Metabolic Disorders) Consensus Conference Statement on One-Anastomosis Gastric Bypass (OAGB-MGB): Results of a Modified Delphi Study. Obes. Surg..

[24] Tacchino R.M. (2015). Bowel length: Measurement, predictors, and impact on bariatric and metabolic surgery. Surg. Obes. Relat. Dis..

[25] Komaei I., Sarra F., Lazzara C., Ammendola M., Memeo R., Sammarco G., Navarra G., Currò G. (2019). One Anastomosis Gastric Bypass-Mini Gastric Bypass with Tailored Biliopancreatic Limb Length Formula Relative to Small Bowel Length: Preliminary Results. Obes. Surg..

[26] Dayan D., Kuriansky J., Abu-Abeid S. (2019). Weight regain following Roux-en-Y gastric bypass: Etiology and surgical treatment. IMAJ.

[27] Kermansaravi M., Shahmiri S.S., Davarpanah Jazi A.H., Valizadeh R., Weiner R.A., Chiappetta S. (2021). Reversal to normal anatomy after one-anastomosis/mini gastric bypass, indications and results: A systematic review and meta-analysis. Surg. Obes. Relat. Dis..

[28] Genser L., Soprani A., Tabbara M., Siksik J.M., Cady J., Carandina S. (2017). Laparoscopic reversal of mini-gastric bypass to original anatomy for severe postoperative malnutrition. Langenbeck’s Arch. Surg..

[29] Hussain A., Van den Bossche M., Kerrigan D.D., Alhamdani A., Par Mar C., Javed S., Harper C., Darrien J., Singhal R., Yeluri S. (2019). Retrospective cohort study of 925 OAGB procedures. The UK MGB/OAGB collaborative group. Int. J. Surg..

[30] Chen C.Y., Lee W.J., Lee H.M., Chen J.C., Ser K.H., Lee Y.C., Chen S.C. (2016). Laparoscopic Conversion of Gastric Bypass Complication to Sleeve Gastrectomy: Technique and Early Results. Obes. Surg..

[31] Kermansaravi M., Mahawar K.K., Davarpanah Jazi A.H., Eghbali F., Kabir A., Pazouki A. (2020). Revisional surgery after one anastomosis/mini gastric bypass: A narrative review. J. Res. Med. Sci..

[32] Boyle M., Mahawar K. (2020). One Anastomosis Gastric Bypass Performed with a 150-cm Biliopancreatic Limb Delivers Weight Loss Outcomes Similar to Those with a 200-cm Biliopancreatic Limb at 18–24 Months. Obes. Surg..

